# Towards applications of genome‐scale metabolic model‐based approaches in designing synthetic microbial communities

**DOI:** 10.15302/J-QB-022-0313

**Published:** 2023-03-01

**Authors:** Huan Du, Meng Li, Yang Liu

**Affiliations:** ^1^ Archaeal Biology Center Institute for Advanced Study Shenzhen University Shenzhen 518060 China; ^2^ Shenzhen Key Laboratory of Marine Microbiome Engineering Institute for Advanced Study Shenzhen University Shenzhen 518060 China

**Keywords:** genome‐scale metabolic modeling, microbial community design, interspecies interaction, environmental impact, community‐level performance

## Abstract

**Background:**

Synthetic microbial communities, with different strains brought together by balancing their nutrition and promoting their interactions, demonstrate great advantages for exploring complex performance of communities and for further biotechnology applications. The potential of such microbial communities has not been explored, due to our limited knowledge of the extremely complex microbial interactions that are involved in designing and controlling effective and stable communities.

**Results:**

Genome‐scale metabolic models (GEM) have been demonstrated as an effective tool for predicting and guiding the investigation and design of microbial communities, since they can explicitly and efficiently predict the phenotype of organisms from their genotypic data and can be used to explore the molecular mechanisms of microbe‐habitats and microbe‐microbe interactions. In this work, we reviewed two main categories of GEM‐based approaches and three uses related to design of synthetic microbial communities: predicting multi‐species interactions, exploring environmental impacts on microbial phenotypes, and optimizing community‐level performance.

**Conclusions:**

Although at the infancy stage, GEM‐based approaches exhibit an increasing scope of applications in designing synthetic microbial communities. Compared to other methods, especially the use of laboratory cultures, GEM‐based approaches can greatly decrease the trial‐and‐error cost of various procedures for designing synthetic communities and improving their functionality, such as identifying community members, determining media composition, evaluating microbial interaction potential or selecting the best community configuration. Future efforts should be made to overcome the limitations of the approaches, ranging from quality control of GEM reconstructions to community‐level modeling algorithms, so that more applications of GEMs in studying phenotypes of microbial communities can be expected.

## Abstract 2

**Author summary:** The applications of computational tools have been demonstrated to increase the development of synthetic microbial communities which is an emerging field and can be used in various biotechnology applications. As one effective tool, genome‐scale metabolic modeling helps to reconstruct testable metabolic networks from genomic information and can quantitatively simulate entire metabolic fluxes of communities in considering microbe‐microbe and microbe‐habitat interactions. In‐depth study of underlying mechanisms of microbial interactions using metabolic models and of coupling the models with multi‐omics data or machine learning can further extend applications in designing synthetic communities.

## INTRODUCTION

Synthetic microbial communities are the basis of an emerging research field in synthetic biology that aims at the in‐detail study of the properties and functions of microbial communities and development of application of these functions in biotechnology. A synthetic microbial community is artificially built by co‐culturing two or more species under controlled conditions [1]. The natural life mode of microorganisms in microbial communities inspires the idea of constructing synthetic communities. Microbes in multi‐species consortia can form “metabolic modules” and accomplish complex metabolic processes via cooperation [2,3]. It may be challenging to observe such a phenomenon with a single strain. For example, when a single engineered *Escherichia coli* strain is used to generate the whole glutarate pathway, the accumulation of the intermediate 5‐AMV limits the glutarate production. While applying a synthetic consortium composed of two engineered *E. coli* strains, which each contribute a part of the glutarate pathway, the inhibition can be totally removed, leading to a 19.2% of improvement in glutarate production [3]. Moreover, the diversity of metabolic capabilities possessed by the multiple species and the inter‐species interactions enhance the stability and robustness of the community against environmental stresses and ecological invasion [4, 5, 6]. Multi‐species consortia can use more kinds of substrates and reduce the inhibition of intermediate products by regulating the populations. Some experiments have also indicated that during the process of biofuel production, assemblages of algae are better than monocultures at resisting contaminations [7]. In recent years, synthetic microbial communities have revealed strong capabilities in various industrial and biotechnological applications, such as environmental remediation [8], chemical production [9], biofuel production [10,11], drug discovery [12], probiotic‐mediated therapies [13,14].

The vast potential of microbial communities is far from harnessed, due to our limited knowledge and ability in rapid design of effective, stable, and robust microbial communities [15]. Among the important challenges for such design are the elusive microbial interactions within the communities [16,17]. Intercellular interactions are one of the key factors for shaping and maintaining community structure [18,19]. Many interactions occur simultaneously to the microbes, including competition for resources and the exchange of metabolites. The tradeoffs led by the interactions make the community composition tend to be stable. In addition, the dynamic variability in interaction patterns can also lead to high metabolic diversity of the communities and hence make the communities more resistant and responsive to environmental perturbations. However, the microbial interactions are extremely complex and difficult to identify. This is due to the high species diversity and hence metabolic diversity of microbes, due to the enormous species of metabolites to be exchanged, due to the multiple promiscuous interactions among microbes, and due to the dynamic changes in interaction patterns in response to environmental conditions.

In this context, the genome‐scale metabolic models (GEMs), which can simulate the metabolic flux distributions of organisms based on their genomic data, offer an effective tool for studying microbial metabolic interactions. Compared to other methods, especially the use of laboratory cultures, GEM‐based approaches can explicitly and efficiently predict and study the underlying molecular mechanisms of the multi‐species interactions as well as the performance of the whole microbial community and their metabolic network [20, 21, 22]. Moreover, the reactions of microbes to different environmental conditions can also be explored, using condition‐specific GEMs. With these prediction capacities, the applications of GEM‐based approaches can greatly decrease the trial‐and‐error cost in various procedures for designing synthetic communities and improving their functionality, overcoming challenges such as identifying community members, determining media composition, evaluating microbial interaction potential or selecting the best community configuration. They should thus be considered indispensable for research on synthetic communities. In the following sections, we first present the fundamentals and the reconstruction process of GEMs. Next, several applications of the GEM‐based approaches for exploring the microbe‐microbe, microbe‐habitat interactions, and the community‐level performance are presented. Finally, the uncertainties of GEM‐based approaches and the future challenges of their applications in the research of synthetic microbial communities are discussed.

## GENOME‐SCALE METABOLIC‐BASED APPROACHES

### What are genome‐scale metabolic models?

A GEM is a mathematical representation of the metabolic network of an organism, which quantitatively predicts its genotype‐phenotype relationship. Using GEMs, a whole optimized set of directional metabolic reactions of a cell can be determined from its genomic data in simulating its entire metabolic flux under preset environmental conditions.

**Figure 1:**
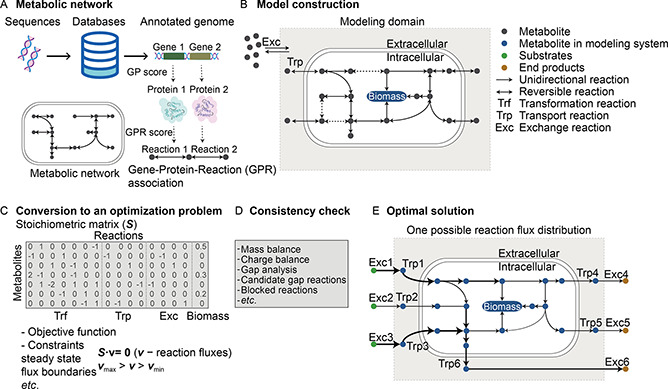
**Basic process of GEM reconstructions.** (A) Metabolic reactions of the organism of interest are derived from the annotated genome depending on the GPR associations. The metabolic network of this organism (schematic representation) is integrated from the set of metabolic reactions. (B) A biomass reaction is added to the metabolic network, and the boundary of the modeling system and the exchange fluxes with the environment are defined for subsequent model construction. (C) The model deals with an optimization problem with an appropriate objective function and several constraints on the reaction fluxes. The stoichiometric matrix converted from the metabolic network delineates the relation between the reactions and their related metabolites and is used to define the key constraint to ensure the steady state of the organism. (D) Consistency check of the metabolic network. (E) One or multiple possible flux distributions can be found by solving the optimization problem.

In the process of GEM reconstruction, the first step is the annotation of the genome sequences using metabolic knowledge bases ( Fig.1), such as the Kyoto Encyclopedia of Genes and Genomes (KEGG) [23], the MetaCyc [24] or the Biochemical, Genetic and Genomic (BiGG) [25] knowledge bases. The annotated genes are associated with their corresponding reactions with a reaction score calculated through gene‐protein‐reaction (GPR) rules. It determines if an adequate collection of proteins is present for catalyzing the reactions ( Fig.1). By deciding the set of biochemical reactions that the organism of interest can carry out, its draft metabolic network can be reconstructed. This draft construction may contain gaps or inaccuracy due to missing or inaccurate gene annotations, which hence should be further revised.

For the model refinement, a biomass reaction [26] is added to this metabolic network to support the cell growth and to connect with the reactions that synthesize precursors for biomass formation ( Fig.1). The biomass reaction involves major compounds that are essential for growth of an organism. Ideally, the biomass formulation of a specific organism, such as *E. coli* [27] and *Methanosarcina barkeri* [28], should be directly determined by experimental measurements. Where there is lack of experimental data, then the biomass composition of template models should be used, such as use of *E. coli* for Gram‐negative bacteria or use of *Bacillus subtilis* for Gram‐positive bacteria [29]. Various GEM construction tools have also built template models for archaea, algae, fungi, plants and human cells [30, 31, 32]. In addition to the biomass formulation, a set of exchange reactions is defined to describe the flux of substrates uptake and end‐products discharge. The new version of the metabolic network can be further converted into a stoichiometric matrix which compiles the stoichiometry information of all the reactions for mathematically linking the metabolites with their related reactions ( Fig.1). The stoichiometric matrix offers analysis of the most important constraints on reaction flux to ensure mass and electron conservation. In addition, other constraints can be set according to thermodynamic feasibility [33] or other experimental results [34]. An objective function ( *e.g.*, the growth rate, or the production rate of a specific metabolite) can be maximized or minimized using mathematical optimization techniques, such as flux balance analysis (FBA) [35], under these constraints. Subsequently, network evaluation is executed to check the consistency of the network, including the check of mass and charge balance, the check of blocked reactions, and the search of candidate reactions for gap filling ( Fig.1). After the network evaluation, the generic GEM can be reconstructed, and the optimal solutions can be quantified to provide possible flux distributions for the metabolic network ( Fig.1). The exchange fluxes describe the uptake and secretion rates of the organism. Generally, the more complex the constraints are, the more precise the reconstructions will be. The environmental conditions, whether as input or as constraints, also greatly affect the flux distributions of reactions in GEMs.

### Genome‐scale metabolic modeling tools

Since the first GEM of *Haemophilus influenzae* Rd was constructed in 1999 [36], genome‐scale metabolic modeling has rapidly developed. Thousands of GEMs have been built for many kinds of cells, such as bacteria [37,38], archaea [39], yeasts [40], plants [41] and even human cells [42]. As the manual process for GEM construction is complex and laborious [26], many computational tools have been developed to make the procedures automatic or semi‐automatic, such as the online tools, ModelSEED [31] and Kbase [43], the canonical modeling toolboxes like the COBRA toolbox [44], and other approaches such as Pathway Tools [45], CarveMe [30], AGORA [46], AGREDA [47], RAVEN [48] or Merlin [49]. These tools greatly accelerate the GEM construction process, leading to generation of an increasing number of GEMs [29]. Moreover, some tools can improve the phenotypic predictions of microbes by incorporating specific constraints, such as enzymatic constraints in GECKO [50] or thermodynamic constraints in PSAMM [51]. In addition, the condition‐specific ( *e.g.*, growth environment, life cycle or specific tissue) metabolisms of organisms can be also simulated based on the generic GEMs and on the experimental observations under specific conditions. These models can help to predict how the microbes allocate nutrients to maximize their growth rate, or their production rate of target chemicals, which further expands the application scope of the GEMs [52, 53, 54, 55].

### Community‐level genome‐scale metabolic modeling tools

**Figure 2:**
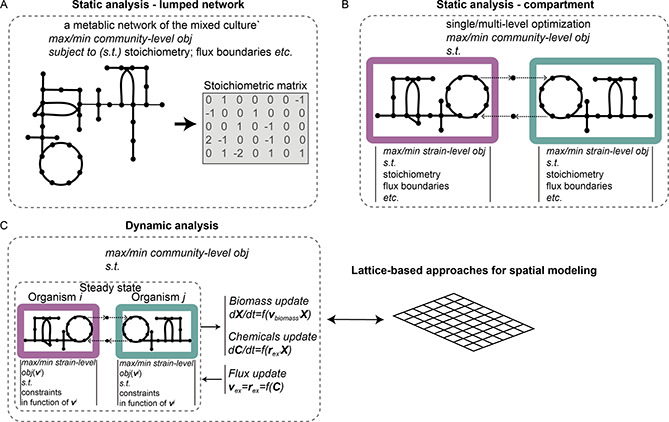
**Different categories of GEM‐based modeling tools.** A) Modeling of the community as a single entity can be achieved by lumped network‐based static analysis that integrates the mixed culture into a unique metabolic network and then converts the network into a whole stoichiometric matrix. (B) Compartment‐based static analysis can model each organism as a distinct compartment in considering the metabolites exchange among them. Multi‐level optimization strategies are executed by adding the sub‐optimization problem for each compartment based on a defined community‐level objective. (C) Dynamic analysis couples the steady‐state modeling with differential equations that capture the temporal variability of community performance. By integrating the dynamic models into a lattice‐based framework, the spatial organization of the community can also be simulated. ***X***, vector denoting the biomass concentration of all the modeled species; ***C***, vector representing all the metabolites concentration; ***v** ^i^*, a subset of all the reaction fluxes of organism *i*; ***v***_biomass_, vector of biomass reaction fluxes of all the modeled species; ***v***_*ex*_, a subset of exchange reaction fluxes of all the modeled species; ***r**_ex_*, a subset of substrates uptake rate of all the modeled species; *t*, time.

Community‐level GEM‐based approaches have been developed by integrating multiple GEMs into one model framework and thereby solving a community‐level optimization problem. One category of the approaches is the static modeling approach that connects species via exchange reactions and assumes a steady state for the whole community. This category can be classified into the lumped network‐based approaches [56,57] that combine the metabolisms of all community members into one network ( Fig.2), and the compartment‐based approaches like OptCom [58], cFBA [59], SteadyCom [21], DOLMN [60], BioLEGO 2 [61] or SMETANA [62] in which each organism is modeled as a distinct compartment and metabolites exchange between these compartments are explicitly modeled ( Fig.2). The compartment‐based approaches can either solve a single objective for the community performance or integrate the species suboptimization simulations into the community‐level optimization. In addition to studying the community‐level performance via the interactions among organisms, the multi‐level optimization can also describe the trade‐offs between individual and community‐level fitness criteria.

Alternatively, dynamic approaches like DMMM [63] or dOptCom [64] can explicitly model the temporal variability of microbial communities. This category couples the static compartment‐based approaches with the differential equations that capture the dynamic variability of modeling components like biomass or metabolite concentrations. By adding the spatial features, some dynamic approaches, including COMETS [65], BacArena [20], IndiMeSH [66], CODY [67] *etc.*, can also predict the spatial heterogeneity of microbial communities ( Fig.2). They hence meet the requirements of the spatio‐temporal control of engineered communities in a structured environment, which cannot be done by static methods.

## APPLICATIONS OF GEM‐BASED APPROACHES IN DESIGNING MICROBIAL COMMUNITIES

**Figure 3:**
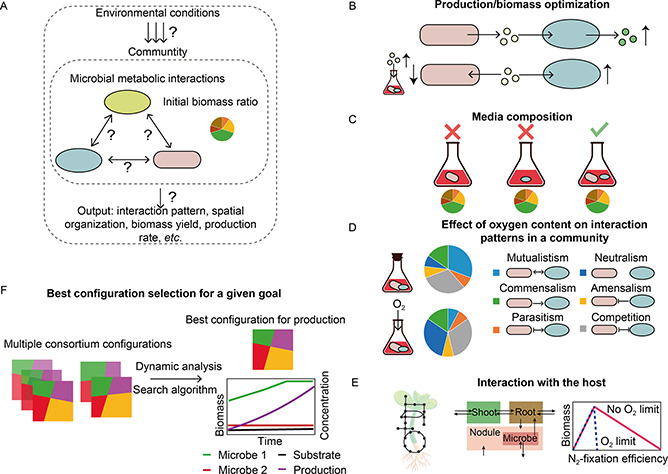
**Examples of GEM applications guiding design of synthetic microbial communities.** (A) Three parts of the GEM applications in studying synthetic microbial communities: microbe‐microbe interactions, environmental impacts, and community‐level performance. (B) Biomass/production optimization by microbial interactions. (C) Optimization of media composition for inducing syntrophic interactions of microbes. (D) Prediction of the effect of oxygen content on all pairwise interactions in a community. (E) The effects of the activity of microbes in the nodules on the biomass production rate of the host plant. (F) Best consortium configuration selection among multiple configurations (including media composition, initial strain ratio, environmental conditions, *etc.*) for a given goal by integrating a dynamic GEM‐based approach with a search algorithm.

GEMs have been developed to study, predict and help to engineer the metabolisms of individual microbes and microbial communities, leading to various direct and indirect applications in systems and synthetic biology [68]. Relevant applications of GEMs guiding design of synthetic microbial communities can be divided into three parts ( Fig.3). Firstly, the microbe‐microbe interactions (positive, neutral, or negative types) should be analyzed. Secondly, it is necessary to consider how environmental fluctuations affect microbial phenotypes and further change microbial interaction patterns. Thirdly, based on the two former studies, the community‐level performance can be predicted and optimized. The GEM‐based approaches mentioned in the three aspects for designing synthetic microbial communities are summarized in Tab.1.

### Studying microbe‐microbe interactions

Modeling metabolic interactions among microbes is one of the most important applications of GEMs. Using GEM‐based approaches, it has been possible to explore underlying mechanisms and predict previously unidentified interactions. The first multispecies GEM was constructed for studying the interaction of *Desulfovibrio vulgaris* and *Methanococcus maripaludis* via a compartmentalized FBA‐based model in which the objective was maximizing a weighted sum of the two‐species biomass production fluxes [69]. The model suggested the essentiality of hydrogen transfer for syntrophic growth and accurately predicted the relative cell proportion of the two species during growth. Unlike that approach [69], the FBA framework named OptCom relied on a multi‐level optimization formulation [58]. This approach considered a separate FBA problem for each species as sub‐optimization problems. It integrated them through the constraints on metabolite exchanges and an outer‐level objective of maximizing community biomass production fluxes. Using OptCom, the metabolic interactions between two abundant species in the human gut, *Bifidobacterium adolescentis* and *Faecalibacterium prausnitzii*, were explored [70]. The modeling results indicated that the butyrate produced by *F. prausnitzii*, which is essential for colonic homeostasis and cancer prevention, can be promoted by the acetate supplied by *B. adolescentis* ( Fig.2). In addition to modeling the positive interactions, other interactions ( *e.g.*, competition, parasitism, *etc.*) could be evaluated using GEM‐based approaches [58,62,71,72]. For example, an approach (SteadyCom) to identifying the competitive relation among microbes was to simulate the relative abundances of the community members and search for negatively correlated pairs while requiring constant community growth rate [21]. The study used four mutant *E. coli* strains for simulations and succeeded in identifying the competitive pairs in which both the strains relied on lysine and methionine.

**Table 1:** Summary of GEM‐based approaches which can be applied in synthetic community researches

Classification	Method	Short description
Community‐level, static, lumped network‐based	Rodríguez *et al*. 2006 [56]	A model to predict product formation from glucose in anaerobic mixed culture fermentation through maximizing a community‐level biomass objective.
	Pramanik *et al*. 1999 [57]	A model to explore biological phosphorus removal metabolism.
Community‐level, static, compartment‐based	OptCom [58]	An FBA‐based framework to describe trade‐offs between individual and community‐level fitness criteria by optimizing multi‐level objectives.
	cFBA [59]	A method to analyze community parameters (maximal growth rate, relative biomass abundance, *etc*.) at balanced growth.
	SteadyCom [21]	A framework reformulated from cFBA without the limitations on the number of linear programming iterations for predicting the variation in species abundance in response to substrate changes.
	DOLMN [60]	A mixed integer linear programming (MILP) optimization approach to explore possible labor division in communities under constraints (*e.g.*, limited number of exchange reactions).
	BioLEGO 2 [61]	A Microsoft Azure Cloud‐based framework which supports large‐scale simulations of biomass serial fermentation processes by two different organisms with single or multiple gene knockouts.
	SMETANA [62]	A tool to estimate pairwise and community‐level microbial interaction potential (through SMETANA score) and identify likely exchanged metabolites.
	Stolyar *et al*. 2007 [69]	The first multi‐species GEM to predict community‐level fluxes and the ratio of cells.
	The microbiome modeling toolbox [71]	A COBRA‐based toolbox to study various types of pairwise microbe‐microbe, microbe‐host interactions and, to analyze personalized gut microbial communities under different diets.
	MMinte [72]	A methodology to assess pairwise microbial metabolic interactions ends the effect of these interactions on the relative growth rates of microbes from 16S rRNA data.
	Klitgord and Segrè 2010 [76]	A model to identify media that can induce putative symbiotic interactions.
	ViNE [81]	An FBA‐based model for analyzing the integrated metabolism of the holobiont consisting of a host plant and its symbiotic bacterium.
	MICOM [86]	A framework for predicting growth rates of diverse bacterial species in human gut and metabolic fluxes of communities by using a heuristic optimization approach based on L2 regularization.
	CASINO [89]	A toolbox for modeling diet‐microbiota interactions.
	Zampieri and Sauer 2016 [94]	A mixed‐integer bi‐level linear programming to infer an optimal combination of nutrients for sustaining pairwise, synergistic growth of microbes with minimum cost of cross‐fed metabolites.
Community‐level, dynamic, temporal	DMMM [63]	The first method using dFBA at community level to optimize growth rates of each strain within the community.
	dOptCom [64]	A method extended from OptCom for the dynamic metabolic modeling of microbial communities with multi‐level objectives.
Community‐level, dynamic, spatio‐temporal	COMETS [65]	A platform implementing a dFBA algorithm on a lattice to track the spatio‐temporal biomass distribution and fluxes of a multi‐species community at population level.
	BacArena [20]	An R package integrating dFBA with individual‐based approach to generate spatial organization and metabolic phenotype in biofilms over time.
	IndiMeSH [66]	A model combined dFBA with individual‐based approach in an angular pore network for spatial modeling of soil aggregates in considering the impact of habitat geometry and hydration conditions.
	CODY [67]	A multi‐scale framework to identify and quantify spatiotemporal‐specific variations of gut microbiome abundance profiles in the colon as impacted by host physiology.
	FLYCOP [100]	A framework combining COMETS with a local search algorithm to automatically select the best consortium configuration among multiple predefined/random ones for a given goal.
Individual level, integration with macromolecular expression	FoldME [78]	A metabolism and protein expression (ME) model incorporating folding and degradation kinetics to predict the effect of temperature on microbial growth.
	OxidizeME [79]	An ME model to describe the response of microbes to reactive oxygen species stress.
	AcidifyME [80]	An ME model integrating folding and unfolding thermodynamics and kinetics to simulate the response of microbes to pH variations.

In addition to analysis of interaction patterns, GEMs have been used to explore the underlying mechanisms for generating interactions among species, which are difficult to assess experimentally and can inform the design of synthetic microbial communities. The impact of costless metabolic secretions was evaluated by performing over 2 million pairwise growth simulations of 24 species in different media [73]. The costless metabolic exchange was indicated to be a driver of beneficial interactions contributing to the better growth of microbes in resource‐poor environments. Anoxic conditions can provide more opportunities for costless metabolic exchanges and more stable ecological network motifs. Another approach named SMETANA assessed the extent of resource competition and metabolic exchanges among microbes via computing the substrate overlap and the essential exchanged metabolites [62]. The simulations for over 800 communities revealed that competition among microbes was apparent in all communities and indicated a significant negative correlation between the competition extent and phylogenetic relatedness of the member species. In addition, no prominent association of co‐occurrence with resource competition was observed. But the metabolic interactions were recorded, leading to the conclusion that metabolic interdependency is a major driver of species co‐occurrence. In turn, this point of view also provided support for mutualistic system constructions.

### Evaluating microbe‐habitat/host interactions

Microbial phenotypes (such as metabolic secretions, growth rate) are significantly related to the environmental conditions, which further affects the interspecies interactions as well as the composition, the stability and even the functions of the whole community. Thus, understanding of the culture conditions, of the potential environmental fluctuations, and of the interactions of microbes with the host are essential for studying and designing synthetic microbial communities. Apart from the interactions among microbes described above, the GEM‐based approaches can also be employed for modeling the phenotypic diversity of microbes – ranging from growth rate and substrate uptake rate to gene expression levels – and hence for predicting diverse interspecies interaction patterns under different environmental conditions [74,75].

In a study, the dynamic multispecies metabolic modeling (DMMM) has been employed to investigate the effect of substrate concentrations on the interactions between *Rhodoferax* and *Geobacter* species, which are both acetate‐oxidizing Fe(III)‐reducers found in uranium‐contaminated groundwater [63]. The model predicted that the high acetate and low ammonium concentrations would increase the ratio of *Geobacter* to *Rhodoferax*, the former of which can help to reduce the uranium in the environment ( Fig.3). This result can provide support for designing strategies for bioremediation of uranium‐contaminated groundwater. Such effort has also been extended to search the media compositions that sustain a co‐culture of two species but do not support the growth of each organism on its own [76]. The results showed that specific media compositions could induce different types of putative symbiotic interactions ( Fig.3). Environmental fluctuations may be more effective than genetic modifications for inducing symbiotic interactions. It further highlighted the crucial effects of environmental conditions on the generation of symbiotic interactions.

Using a GEM‐based approach, oxygen availability was also found to be able to change microbial interaction patterns. Heinken and Thiele [77] used GEMs for 11 representative gut microbes to model pairwise interactions under anoxic and normoxic conditions ( Fig.3). The mutualistic behaviors of the probiotic organism *Lactobacillus plantarum* towards six other species under anoxic conditions were found to be entirely abolished under normoxic conditions. Furthermore, by incorporating transcription, translation, and stress response mechanisms into GEMs, the metabolism, the proteomic allocation, and the protein folding rates can be modeled. This enabled modelling of cellular behaviors in more detail and led to investigation of the responses of microbes to other environmental perturbations, such as the thermal [78], oxidative [79], and low‐pH stress [80].

The interaction between the microbe and the host is also an important topic that can be studied using GEMs. For example, a model called ViNE integrated the host *Medicago truncatula* (plant) and its symbiotic bacterium *Sinorhizobium meliloti* into a three‐tissue (shoot, root, and nodule) framework to study their association patterns [81] ( Fig.3). The analysis revealed diminishing returns in terms of plant growth when the nitrogen fixation efficiency or the nodulation rate of the bacteria was beyond the optimum, which may have implications for engineering symbiotic nitrogen fixation. Another research direction concerns the metabolic interplay between the host and the gut microbiome, which has been demonstrated to be clearly associated with human health and diseases [82, 83, 84]. GEM‐based approaches were developed to study the effect of the microbiota on the host and also the impact of diet on the gut microbiome [85, 86, 87, 88, 89]. One of the common toolboxes is CASINO, used in a diet‐intervention study of 45 obese human individuals [89]. That study estimated the metabolic capabilities of the gut microbes and successfully predicted a significant change in the levels of some short‐chain fatty acids and amino acids in response to the dietary intervention. Since abundant GEMs have been systematically constructed specifically for study of the members of the gut microbiome [46,47] and human cells [90, 91, 92, 93], we believe that the study on the metabolic exchanges between the microbes, lumen and human cells can be further improved.

### Community‐level performance: design and optimization

Apart from exploring the underlying mechanisms of microbial interactions, several GEM‐based approaches can be employed to model the performance of the whole microbial community and even design or optimize synthetic microbial communities. Here, we provide some examples of applications that use both static and dynamic approaches.

The static approaches can be applied in designing synthetic communities, in various ways. For instance, OptCom can assess the level of sub‐optimal growth in microbial communities [58]; SteadyCom focuses on predicting the variation in species abundance in response to substrate changes [21]; several ad‐hoc approaches can optimize medium composition to induce microbial interactions [76,94]; SMETANA is used to evaluate the extent of resource competition and metabolic interaction potential of a whole community [62]. In particular, in a study integrating SMETANA with a network analysis method, a “social” network for a community was constructed based on the pairwise interaction potential of all the community members in mangrove sediments [95]. According to the network analysis and the transcriptomic data, several microbial active functional modules (mAFMs) were extracted from the network as the core modules. The microbes possess relatively high metabolic interactions and can actively realize certain dominant functions in element transformations via cooperation. These mAFMs represent the sub‐consortia composed of microbes that are highly associated through their positive interactions, their simultaneously high‐level transcriptional activity, and their spatial clustering. They hence could provide clues for synthetic community compositions. In addition, DOLMN has been applied to simulate the trade‐off between metabolic self‐reliance and mutualistic exchange and to further optimize the strategies for metabolic division of labor in ways that would be difficult to identify manually [60]. The simulations for consortia combined with diverse strains of *E. coli* indicated the nuanced and nonintuitive division of labor, like splitting the tricarboxylic acid (TCA) cycle into two separate halves.

Alternatively, dynamic approaches are more suitable for modeling the impact of the spatial heterogeneity of the media distribution or structured environments on microbial communities. By implementing a dynamic FBA algorithm [96] on a lattice, COMETS realized the simulation of the spatial and temporal diffusion of microbial populations and metabolites [65]. By comparing predictions with the experimental results, this model was verified to be able to precisely predict the impact of a competitor on the growth of a two‐species consortium and the spatial distribution of the metabolites’ concentrations. Approaches subsequent to COMETS have made various changes. For example, BacArena, which incorporated GEMs into an agent‐based approach, described the individual cells in more details by modeling heterogeneous phenotypic behavior, like cell movement, replication, or cellular lysis [20]; IndiMeSH adapted the model to study microbial dispersion and nutrient diffusion in more complex habitats such as soil, including pore spaces and aqueous phase configurations [66]. Another approach termed CODY [67] was constructed based on elementary flux mode analysis instead of dynamic FBA. CODY focused on modeling gut microbiota and hence integrated three multiscale modeling components, *i.e.*, species‐level microbial dynamics, microbial interactions, and colon physiology. This framework has enabled spatiotemporal predictions of microbial variations in response to diet intervention. In addition, some dynamic analyses have been used to optimize desired community‐level functions, such as maximizing ethanol production with *S. cerevisiae* and *E. coli* [97,98] or to improve bioprocessing of cellulose with a clostridial consortium [99].

Going further than the tools for investigating some of the factors involved in constructing synthetic communities, a framework termed FLYCOP was developed to directly engineer microbial communities [100]. This framework combined COMETS with a local search algorithm, rather than tuning each control point for designing synthetic communities one by one, automatically selected the best consortium configuration among multiple predefined or random alternatives for a given goal ( Fig.3). FLYCOP could realize various applications, like detecting limiting nutrients, optimizing cross‐feeding relationships, optimizing strain ratios and pathway fragmentation, identifying aerobic‐anaerobic switching time. One example was to optimize a *Synechococcus elongatus‐Pseudomonas putida* consortium to produce the maximum amount of bioplastic. The simulations proposed the best configuration parameters related to initial low NH_4_ concentration and high *S. elongatus* biomass ratio.

## PERSPECTIVE AND FUTURE DIRECTIONS

Since the 1990s, the GEMs and the GEM‐based modeling approaches have achieved rapid progress and have been applied in various fields [29,101]. Notably, GEM‐based approaches have been demonstrated to be indispensable for studying systems and synthetic biology, due to their capacity to predict the genotype‐phenotype relationships of organisms [102]. Engineering individual strains, like targets prediction for gene manipulation, has achieved great successes in various applications with the aid of GEM‐based approaches [103, 104, 105, 106]. Compared with individual cell studies, although in the infancy stage, a growing number of GEM‐based approaches belonging to two categories, the community‐level steady‐state analysis and dynamic framework for spatio‐temporal predictions, have been developed to explore the emergent properties of microbial communities. These GEM‐based frameworks can contribute to giving mechanistic insights into community‐level complexity as well as to improving the design of synthetic microbial communities that are laborious and even impossible to study by way of laboratory experiments. Such beneficial uses include exploring the core modules of a complex community [95] or optimizing the media composition by testing a large number of component combinations [76].

**Figure 4:**
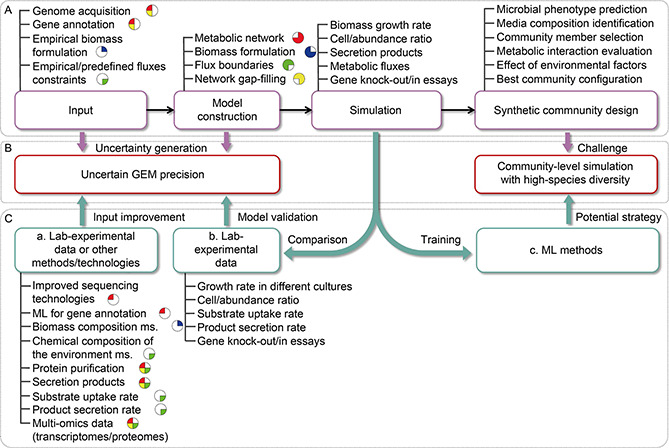
**Limitations (B) hindering the application of GEM‐based approaches in synthetic community design (A) and the potential improvement strategies (C).** (A) An outline summary describing the necessary input data to the corresponding model construction processes (with correspondence represented in the pie chart by the same color), the possible simulated results and the potential applications of GEM‐based approaches in synthetic community design. (B) The bottlenecks are mainly caused by the uncertainties generated during GEM constructions and the challenge of community‐level simulation with highspecies diversity. (C) The potential strategies for improving GEM precision (a, b) and for enabling high‐species simulations (c). a. The data obtained from wet lab‐experiments, machine learning (ML) systems and other methods/technologies can be directly integrated into different processes of GEM construction (with correspondence of the improvement, based on data, to the model construction processes being represented in the pie chart by the same color); b. The GEMs can be validated by comparing the simulated results to the wet lab‐experimental observations; c. Some ML systems trained with the simulated results of GEMbased approaches may explore the underlying interaction mechanisms of the community with high‐species diversity for synthetic community design. ML, machine learning; ms., measurement.

However, the uncertain precision of GEMs and the limited capacity for community‐level simulations with high‐species diversity of current GEM‐based approaches hinder the scope and precision of their applications in the field of synthetic microbial communities ( Fig.4). Basically, the genome sequences, the gene annotations, the biomass formulation and the constraints on metabolic fluxes, which can be obtained from experiments and/or databases, are essential for the construction of GEMs and GEM‐based community‐level models ( Fig.4). The uncertainties that emerge from these data, during the different steps of the GEM reconstruction process [107], such as incorrect/missing gene annotations, lack of specific biomass formulation, unknown media uptake rates, or biased flux simulation, fundamentally limit the precision of the GEM reconstructions and hence affect the community‐level predictions. For example, after swapping biomass formulations between five different bacterial GEMs, considerable changes (up to 32.8%) have been observed in essentiality predictions of reactions, indicating the great impact of the biomass formulation selection on the prediction capacity of GEMs [108]. The precision of GEMs is one of the reasons why the application examples of the most GEM‐based approaches have been executed with communities formed by precisely curated GEMs such as those reconstructed for *E. coli* strains. Thus, the first important task for modeling microbial communities is to certify the quality of GEMs. Recent research has proposed a tool termed MEMOTE for benchmarking GEMs from annotation and basic tests for model components like GPR rules, biomass reaction, and stoichiometry, which significantly contribute to standardized quality control of GEMs [109].

In addition, future efforts should be made to reduce the uncertainties in the GEM reconstruction process and improve GEM precision directly ( Fig.4). It is firstly essential to utilize efficient methods for providing high‐quality genomes to provide the basis for high‐quality GEM reconstructions, such as long‐read sequencing technologies [110] or droplet‐based microfluidics methods [111]. The quality of the assembled genomes can greatly affect the accuracy of the corresponding GEM reconstruction, especially while modeling complex communities as in the gut or soil microbiome. The biomass formulation of a given organism should be estimated more accurately, for instance by lab‐culture measurement or by using the software BOFdat [112]. Moreover, some other experimental observations, such as chemical composition of the microbial habitats, substrate uptake/product secretion rate, multi‐level (individual/community) growth rate, species abundance ratio or gene knock‐out essays, can either be directly integrated as flux constraints of the related reactions in GEM reconstructions or be used to validate the GEMs [37,113,114]. Integrating multi‐omics data, like transcriptome, proteome, or metabolome, into GEMs can also propose more constraints via setting thresholds related to gene/protein expression level or enzymatic activities, to reduce the flux variabilities. Nevertheless, the lack of kinetic information and the high computational demands of this framework result in the challenge for their applications, especially for multi‐species modeling. In this context, recent studies have combined machine learning (ML) methods with GEMs to improve the prediction precision of the genotype‐phenotype relationship with low computational costs. The ML methods can, on the one hand, be applied to decrease the uncertainties in GEM reconstruction processes. For example, the ML systems can be trained to improve gene annotation precision with the gold standard dataset covering more than 1 million protein sequences and their EC numbers [115]. The important reactions for further manual curation can also be identified by an ML system trained with an ensemble of GEMs generated from a draft GEM by iterative gap‐filling [116]. An ML system trained with experimental data consisting of the phenotypic outcomes from single knockout mutants can accurately predict the essentiality of reactions [117]. On the other hand, ML systems can integrate the fluxomic data, generated using GEMs under different growth conditions, with other omics data to reversely improve the prediction power of GEMs, such as in the assessment/improvement of microbial growth and bio‐production [118, 119, 120], in the exploration of antibiotic efficacy [121], or in the prediction of drug targets [122].

Even if the quality of each single‐strain GEM can be accurately reconstructed, particular challenges will still hamper progress in community‐level metabolic modeling. Current modeling tools, except the lumped network‐based approaches, have not been used to simulate complex communities with high species diversity due to the combinatorial complexity of the multi‐level optimizations. For this task, an ML random forest method combined with a dynamic GEM‐based approach has classified the interactions and globally predicted the highly interaction‐related metabolites for a 100‐species gut microbiome [123] ( Fig.4). Another study trained support vector machine models with over 2 million GEM‐generated pairwise simulations to quantify the impact of several conditional variables, such as oxygen availability, species identity and carbon source types, on the secretion of costless metabolites which may promote inter‐microbial interactions [73]. Nevertheless, these frameworks focused on the performance of microbial interactions but cannot directly model the growth and metabolites secretions of a whole community. More work is needed in the future for direct community‐level metabolic modeling for large communities. In addition, it would also be interesting to extend the application of GEM‐based tools to the large space‐scale or even the three‐dimensional organization of microbial communities for modeling microbial aggregations like biofilms for chemical production, granules for wastewater treatment, or other microbial growth in structured environments. Ultimately, this paper shows that tools that can systematically engineer microbial communities are still scarce. Different strategies have distinct focuses regarding environment specification [67], implementation conditions, and outcome results [124]. Thus, while performing synthetic consortia modeling, attention should be paid to selecting an appropriate GEM‐based tool depending on the modeling purpose, the assumption consistency, and the available data.

Overall, the GEM‐based approaches can guide the design of synthetic microbial communities in various ways, including by optimizing community composition, media composition, culture conditions, microbial interactions, and community‐level perturbations under host/habitat condition change. With the advance of omics‐data techniques and the emergent strength of integrating multiple computational methods like GEMs with machine learning, GEM‐based approaches exhibit an extending scope of applications. However, future efforts should be made to overcome the limitations so that more applications of GEMs in studying microbial interactions can be expected.

## COMPLIANCE WITH ETHICS GUIDELINES

The authors Huan Du, Meng Li, Yang Liu declare that they have no conflict of interest or financial conflicts to disclose.

This review does not contain any studies with human or animal subjects performed by any of the authors.
